# Systemic antifungal strategies in allogeneic hematopoietic stem cell recipients hospitalized in french hematology units: a post-hoc analysis of the cross-sectional observational AFHEM study﻿

**DOI:** 10.1186/s12879-022-07216-6

**Published:** 2022-04-09

**Authors:** Mauricette Michallet, Jean El Cheikh, Raoul Herbrecht, Ibrahim Yakoub-Agha, Denis Caillot, Jean-Pierre Gangneux

**Affiliations:** 1 Service d’Hématologie Clinique, Centre Léon Bérard, 28 Rue Laennec, 69008 Lyon, France; 2 Unité de Transplantations et Thérapies Cellulaires, Institut Paoli Calmettes, Marseille, France; 3 Department of Oncology and Hematology, Hôpitaux Universitaires de Strasbourg and Université de Strasbourg, Inserm, UMR-S1113 / IRFAC, Strasbourg, France; 4 LIRIC INSERM U995, CHU de Lille, Université Lille 2, Lille, France; 5 Service d’Hématologie Clinique, Hôpital du Bocage, Dijon, France; 6 Service de Parasitologie- Mycologie, CHU de Rennes, Rennes, France; 7 Present Address: Bone Marrow Transplant Unit, American University of Beirut Medical Center, Beirut, Lebanon

**Keywords:** Allogeneic hematopoietic stem cell transplantation, Antifungal stewardship, Invasive fungal diseases, Prospective, observational study, Prophylaxis

## Abstract

**Background:**

Invasive fungal diseases (IFD) remain a major complication of allogeneic hematopoietic stem cell transplantation (alloHSCT) and are associated with high mortality rates in patients receiving alloHSCT. Antifungal prophylaxis is increasingly being used in the management of IFDs in patients receiving alloHSCT.

**Methods:**

A post-hoc analysis of the cross-sectional observational AFHEM study was carried out to describe the use of antifungal drugs in real-life clinical practice in alloHSCT recipients hospitalized in French hematological units.

**Results:**

A total of 147 alloHSCT recipients were enrolled; most were adults (*n* = 135; 92%) and had received alloHSCT < 6 months prior to enrollment (*n* = 123; 84%). Overall, 119 (81%) patients received a systemic antifungal therapy; of these, 95 (80%) patients received antifungal prophylaxis. Rates of patients receiving systemic antifungal treatment were similar irrespective of transplant time, neutropenic, and graft-versus-host disease status. Among patients on systemic antifungal treatment, 83 (70%) received an azole, 22 (18%) received an echinocandin, and 16 (13%) received a polyene.

**Conclusions:**

This work provides evidence of the antifungal strategies used in alloHSCT recipients hospitalized in French hematological units. Unlike earlier studies, the AFHEM study showed that prophylaxis appears to be the leading antifungal strategy used in alloHSCT recipients in France.

## Background

Invasive fungal diseases (IFDs) remain a major complication of allogeneic hematopoietic stem cell transplantation (alloHSCT). IFDs are mostly life threatening, and an early diagnosis and initiation of appropriate antifungal therapy are essential for improving the clinical outcome [1]. Invasive candidiasis and invasive mold infections cause significant morbidity in the transplant population, especially in those undergoing alloHSCT [2, 3]. Despite the introduction of broad-spectrum antifungal agents over the past two decades, the mortality risk associated with IFDs in alloHSCT recipients remains high [4], with many factors affecting the risk of IFD in these patients including duration and severity of myelosuppression and immunosuppression, severe acute or chronic graft-versus-host disease [GVHD]-particularly if on long-term corticosteroids [5, 6]. The overall incidence of IFDs among high-risk alloHSCT recipients ranges from 10 to 25% [6–9].

The management of IFDs is increasingly moving towards prophylaxis. The use of antifungal agents under real-world conditions of medical practice in a cross-sectional, observational study that examined IFD management in pediatric and adult hematologic units in France (AFHEM), showed that systemic antifungal prophylaxis was used in 76% of patients in this setting [10]. The most commonly used antifungal agents in the AFHEM study were fluconazole and posaconozole [10]. Other antifungal agents are demonstrating efficacy in prophylaxis, offering alternatives to azoles, such as fluconazole and posaconazole. The echinocandin, micafungin, an inhibitor of fungal cell wall β-glucan synthesis with potent activity against most *Aspergillus* and *Candida* species, was used successfully in prophylaxis in patients undergoing haplo-identical hematopoietic stem-cell transplant [11], and in those with hematological malignancies [12]. In both settings, micafungin prophylaxis was well tolerated with few treatment-related adverse events [11, 12].

Within a context where both treatments and guidelines are rapidly evolving, this post-hoc, subgroup analysis of the AFHEM study aimed to assess the management of IFDs in alloHSCT recipients in France. Additionally, this study aimed to describe the frequency of systemic antifungal treatment used according to different strategies (e.g. prophylaxis, empiric, pre-emptive or curative) and to assess the characteristics of alloHSCT recipients receiving these treatments. It also evaluated the prescription modalities used in clinical practice within this specific population, which should benefit from antifungal stewardship programs.

## Methods

### Study design

This was a post-hoc study, analyzing data collected previously by the AFHEM study, a French, prospective, observational, cross-sectional study conducted between September 16, 2013 and October 25, 2013 [10]. French hematological units of university hospital and cancer medical centers were invited to participate. The study was conducted in accordance with the Declaration of Helsinki and with Good Epidemiological Practices. Approvals from national review boards (Comité Consultatif sur le Traitement de l’Information en matière de Recherche dans le domaine de la Santé and Commission Nationale de l’Informatique et des Libertés) were obtained. Patients hospitalized in participating units were required to provide written informed consent prior to inclusion in the study. The 2008 EORTC/MSG criteria were used to define IFDs [13]. No other selection criteria were applied.

### Data collection

Prior history of IFDs, hematological malignancies and underlying conditions, transplant procedures, antifungal treatments, and other ongoing treatments were collected for all patients. Clinical signs, imaging, and other examinations related to IFD episodes were collected for patients who received pre-emptive or curative treatment. If applicable, IFD classification was established at the physician’s discretion. All data were recorded through a unified secure online case-report form.

### Statistical analyses

The present analysis is a post-hoc analysis focusing on alloHSCT recipients. Subgroup analyses were performed according to the systemic antifungal therapeutic strategy (i.e., prophylactic antifungal treatment, empiric antifungal treatment, pre-emptive or curative antifungal treatment) and according to alloHSCT timing (i.e., alloHSCT performed < 6 months or ≥ 6 months prior to the study). Quantitative variables were analyzed in terms of mean, standard deviation, first quartile, median, third quartile, minimum, and maximum. Qualitative and ordinal variables were analyzed in terms of number and frequency within each modality. Frequencies were presented with the associated 95% confidence intervals calculated according to Wilson’s method (with continuity correction). All analyses were carried out using Statistical Analysis Software (SAS), version 9.2 (SAS Institute Inc., Cary, North Carolina, USA).

## Results

### Participating centers

Twenty-four hematological centers in France participated in the study, of which 20 contributed with patients undergoing alloHSCT. Eighteen of these centers were university hospitals and the remaining two were cancer medical centers.

### Patients

A total of 494 patients were included in the cross-sectional AFHEM study, of which 147 alloHSCT recipients were included in this post-hoc analysis. Patient characteristics are summarized in Table 1.

**Table 1:** Characteristics of patients according to transplant anteriority

Characteristic	alloHSCT anteriority		
	< 6 months (*n* = 123)	≥ 6 months (*n* = 24)	Total (*N* = 147)
Sex: male	70 (57)	9 (38)	79 (54)
Median age (range), years	50.2 (0.1–71.2)	48.2 (3.0–68.0)	49.8 (0.1–71.2)
*Hematological malignancy*			
Acute myeloid leukemia	56 (46)	11 (46)	67 (46)
Myelodysplastic syndrome	7 (6)	1 (4)	8 (5)
Acute lymphoblastic leukemia	18 (15)	7 (29)	25 (17)
Hodgkin lymphoma	12 (10)	1 (4)	13 (9)
Non-Hodgkin lymphoma	11 (9)	0	11 (7)
Chronic lymphoid leukemia	1 (1)	0	1 (1)
Myeloma	6 (5)	3 (13)	9 (6)
Chronic myeloid leukemia	6 (5)	0	6 (4)
Other	6 (5)	1 (4)	7 (5)
Disease status: relapse or refractory	71 (58)	21 (88)	92 (63)
*Underlying conditions*			
GVHD^a^	34 (28)	13 (54)	47 (32)
Grade I–II acute GVHD	18 (15)	5 (21)	23 (16)
Grade III–IV acute GVHD	14 (11)	4 (17)	18 (12)
Chronic GVHD	4 (3)	9 (38)	13 (9)
Neutropenic phase	52 (42)	12 (50)	64 (44)
Persistent fever refractory to antibiotic therapy	13 (11)	2 (8)	15 (10)
Previous IFD	26 (21)	7 (29)	33 (22)
Proven IFD Probable IFD Possible IFD	2 (2) 4 (3) 2 (2)	0 (0) 2 (8) 1 (4)	2 (1) 6 (4) 3 (2)
*Ongoing treatments*			
Antibiotics	104 (85)	21 (88)	125 (85)
Immunosuppressors	91 (74)	11 (46)	102 (69)
Antivirals	112 (91)	18 (75)	130 (88)
*Time since entry in the unit*			
≥ 30 days	34 (28)	3 (13)	37 (25)
< 15 days	68 (55)	17 (71)	85 (58)
Median time, days (range)	12 (0–143)	6.5 (0–56)	11 (0–143)
Hospitalization in a room with air treatment	108 (88)	19 (79)	127 (86)
Laminar flow sterile room or IMMUNAIR™ bed^b^	85 (79)	11 (58)	96 (76)
Highly purified HEPA-filtered room^b^	19 (18)	5 (26)	24 (19)
Conventional room^b,c^	4 (4)	3 (16)	7 (5)

Data are *n* (%), unless otherwise specified

alloHSCT: allogeneic hematopoietic stem cell transplantation; GVHD: graft-versus-host disease; HEPA: high-efficiency particulate air; IFD: invasive fungal disease

^a^Chronic and/or acute GVHD

^b^Percentage among patients hospitalized in room with air treatment

^c^Room with PLASMAIR™ or equivalent

Most patients (*n* = 135; 92%) were adults and the median age was 49.8 years (range 0.1–71.2). Among 147 alloHSCT patients, the major indications were acute leukemia for 92 patients (63%) and lymphoma for 24 patients (16%). At the time of the study, 92 (63%) patients were in relapse or had refractory disease. In total, 130 (88%) patients were receiving antivirals at the time of inclusion, 125 (85%) received antibiotics, 102 (69%) received immunosuppressive agents, and 63 (43%) received chemotherapy (including conditioning regimen). Less than a quarter of patients (*n* = 33; 22%) had a previous history of IFD, of which two (1%) had proven IFD, six (4%) had probable IFD and 3 (2%) had possible IFD according to EORTC/MSG criteria [13]. Eighty-five patients (58%) had been hospitalized for < 15 days and only 20 (14%) had been placed in a conventional room without air treatment. The remaining 127 (86%) patients were either hospitalized in a laminar flow room or IMMUNAIR™ bed (*n* = 96; 76%), or in a highly purified high-efficiency particulate air (HEPA)-filtered room (*n* = 24; 19%), or in a conventional room with PLASMAIR™ (*n* = 7; 5%).

After alloHSCT, 47 (32%) patients had developed GVHD, mainly acute GVHD (*n* = 41; 28%), and 64 (44%) patients were neutropenic. Most patients (*n* = 123; 84%) had undergone alloHSCT < 6 months previously. Of these, 95 (77%) were in the pre-engraftment period (i.e., within 2 months from conditioning regimen); 66 of which were at high risk of IFD according to the European Conference on Infections in Leukemia-5 (ECIL-5) definition [14].

Patients’ characteristics were different when split according to transplant timing (Table 1). In the group of patients who received alloHSCT < 6 months prior to the study (*n* = 123), 23 (19%) patients had lymphoma (either Hodgkin lymphoma or non-Hodgkin lymphoma) versus one patient (4%) in the group of patients transplanted ≥ 6 months prior to the study (*n* = 24). With regard to conditions of hospitalization, the median time of hospitalization at the time of enrollment in the study was 12 days (range 0–143) for patients who received alloHSCT < 6 months prior to the study and 6.5 days (range 0–56) for patients who received alloHSCT ≥ 6 months prior to the study. As expected, most patients (*n* = 21; 88%) who received alloHSCT ≥ 6 months prior to the study were in the relapse or refractory phase, compared with 71 (58%) in the group that received alloHSCT more recently. More than half of the patients (54%) who received alloHSCT ≥ 6 months prior to the study (*n* = 13) presented with acute and/or chronic GVHD, compared with 28% (*n* = 36; mostly with acute GVHD) of patients who received alloHSCT more recently. It should also be noted that seven out of 24 patients (29%) who received alloHSCT ≥ 6 months prior to the study had already experienced a previous IFD, compared with 26 out of 123 patients (21%) who received alloHSCT more recently. Of the patients that had undergone alloHSCT < 6 months previously (*n* = 123; 84%), two patients had proven IFD, four probable IFD and two possible IFD, according to the EORTC/MSG criteria [13]. Of the patients that had undergone alloHSCT ≥ 6 months prior to the study (*n* = 24; 16%), none had proven IFD, two had probable IFD and one had possible IFD. Of the 123 patients who received alloHSCT more recently, 91 (74%) were receiving immunosuppressive treatment compared with 11 (46%) who received alloHSCT ≥ 6 months prior to the study. The proportion of patients in the neutropenic phase was similar between the two groups: *n* = 52 (42%) and *n* = 12 (50%) in patients who received alloHSCT < 6 months and ≥ 6 months prior to the study, respectively. However, it was assumed that the group of patients who received transplantation more recently were mostly in the pre-engraftment phase, while those belonging to the other group were in the late neutropenic phase, probably related to a relapse or to chronic GVHD and its treatment.

### Antifungal strategies

The frequency of systemic antifungal treatments used are summarized in Tables 2 and 3. In total, 119 (81%) patients received a systemic antifungal treatment (as part of a prophylactic, empiric, pre-emptive, or curative strategy) during at least one of the 5-day observational periods (Table 2). More patients received systemic antifungal treatment in the group who received alloHSCT ≤ 2 months prior to the study (*n* = 85; 89%) compared with those who received alloHSCT < 6 months and ≥ 6 months prior to the study (*n* = 17 [61%] and *n* = 17 [71%], respectively). Neutropenic phase and GVHD status had no impact on the frequency of systemic antifungal treatment. Overall, 95 (65%) patients received antifungal prophylaxis, representing 80% of patients treated with antifungals. Of these patients, 76 (80%) received primary antifungal prophylaxis and 19 (20%) received secondary antifungal prophylaxis. Thirteen (9%) patients received antifungal empiric therapy and 11 (7%) patients received antifungal pre-emptive or curative therapy. The rates of patients who received systemic antifungal treatment were similar, regardless of the timing of the transplant, neutropenic status, and GVHD status. Nevertheless, the rate of patients treated with systemic pre-emptive or curative antifungal therapy was higher in patients who had GVHD (either chronic or acute) (*n* = 6 [17%]) and received alloHSCT ≥ 6 months prior to the study (*n* = 3 [18%]), compared with patients with no GVHD (*n* = 5 [6%]) and patients more recently transplanted (*n* = 8 [7%], respectively) (Table 2). Considering patients in the pre-engraftment period and patients developing GVHD, most (*n* = 85; 89%) had received systemic antifungal therapy, especially those presenting with a high-risk profile (acute myeloid leukemia, refractory or relapsed acute lymphoblastic leukemia, age over 65, history of IFD, and acute GVHD) (Table 3).

**Table 2:** Frequency of systemic antifungal strategies used, according to transplant anteriority, neutropenic phase, and GVHD status

	alloHSCT timing			Neutropenia		GVHD^a^		Total
	≤ 2 months (*n* = 95)	> 2–<6 months (*n* = 28)	≥ 6 months (*n* = 24)	No (*n* = 83)	Yes (*n* = 64)	– (*n* = 100)	+ (*n* = 47)	(*N* = 147)
Not treated with systemic antifungals	10 (11)	11 (39)	7 (29)	15 (18)	13 (20)	17 (17)	11 (23)	28 (19)
Treated with systemic antifungals	85 (89)	17 (61)	17 (71)	68 (82)	51 (80)	83 (83)	36 (77)	119 (81)
Prophylaxis^b^	68 (80)	14 (82)	13 (76)	57 (84)	38 (75)	68 (82)	27 (75)	95 (80)
Empiric strategy^b^	11 (13)	1 (6)	1 (6)	4 (6)	9 (18)	10 (12)	3 (8)	13 (9)
Pre-emptive or curative strategy^b^	6 (7)	2 (12)	3 (18)	7 (10)	4 (8)	5 (6)	6 (17)	11 (7)

Data are *n* (%)

alloHSCT: allogeneic hematopoietic stem cell transplantation; GVHD: graft-versus-host disease

^a^Acute and/or chronic extensive

^b^Percentage among patients treated with systemic antifungals

**Table 3:** Frequency of systemic antifungal strategies used, according to the pre-engraftment profile (*N* = 95)

	Pre-engraftment low risk (*n* = 29)	Pre-engraftment high risk^a^ (*n* = 54)	GVHD^b^ (*n* = 12)	Total (*N* = 95)
Not treated with systemic antifungals	4 (14)	4 (7)	2 (17)	10 (11)
Treated with systemic antifungals	25 (86)	50 (94)	10 (83)	85 (89)
Prophylaxis^c^	22 (88)	39 (78)	7 (70)	68 (80)
Empiric strategy^c^	3 (12)	7 (14)	1 (10)	11 (13)
Pre-emptive or curative strategy^c^	0	4 (8)	2 (20)	6 (7)

Data are *n* (%)

GVHD: graft-versus-host disease

^a^With risk factors other than GVHD

^b^Patients presenting with GVHD grade II, III, or IV

^c^Percentage among patients treated with systemic antifungals

### Antifungal drugs

Prescribed antifungal regimens and strategies are summarized in Table 4. Overall, 83 (70%) patients who received systemic antifungal treatment received an azole, 22 (18%) an echinocandin, and 16 (13%) a polyene (mostly intravenous liposomal amphotericin B). The most commonly used prophylactic drugs were fluconazole (administered to 44 [46%] patients who received prophylaxis), posaconazole (*n* = 16; 17%), amphotericin B formulations (*n* = 11; 12%), and caspofungin (*n* = 11; 12%). Regarding empiric therapy, nine (69%) patients received caspofungin, two (15%) patients received liposomal amphotericin B, and the remaining two (15%) patients received an azole (either oral fluconazole or intravenous voriconazole). When curative or pre-emptive therapy was used, voriconazole was the most common antifungal drug (*n* = 8; 73%); most of these patients (*n* = 6) received oral voriconazole.

**Table 4:** Summary of systemic antifungal agents administered during the 5-day observation period

	Prophylaxis (*n* = 95)	Empiric strategy (*n* = 13)	Pre-emptive or curative strategy (*n* = 11)	Total (*N* = 119)
Missing values	1 (1)	0	0	1 (1)
Azoles alone	70 (74)	2 (15)	8 (73)	80 (67)
Polyenes alone	10 (11)	2 (15)	1 (9)	13 (11)
Echinocandins alone	13 (14)	9 (69)	0	22 (18)
Polyenes and azoles	1 (1)	0	2 (18)	3 (25)
*Azole type*				
* N*^*a*^	71	2	10	83
PO fluconazole	38 (54)	1 (50)	0	39 (47)
IV fluconazole	6 (8)	0	0	6 (7)
PO posaconazole	16 (23)	0	2 (20)	18 (22)
PO voriconazole	8 (11)	0	6 (60)	14 (17)
IV voriconazole	3 (4)	1 (50)	2 (20)	6 (7)
*Polyene type*				
* N*^*a*^	11	2	3	16
IV liposomal amphotericin B	8 (73)	2 (100)	3 (100)	13 (81)
IV conventional amphotericin B	3 (27)	0	0	3 (19)
*Echinocandin type*				
* N*^*a*^	13	9	0	22
Caspofungin	11 (85)	9 (100)	0	20 (91)
Micafungin	2 (15)	0	0	2 (9)

All data are *n* (%)

IV: intravenous; PO: per os (orally)

^a^Percentages for each antifungal drug are based on the *N* values for each drug subcategory

### Characteristics of patients receiving antifungal therapy

Characteristics of the alloHSCT recipients who received different systemic antifungal treatments are summarized in Table 5. A total of 102 (86%) patients received alloHSCT < 6 months prior to the study and 85 (71%) patients were in the pre-engraftment phase (i.e., within 2 months from receiving a conditioning regimen). Most patients (*n* = 105; 88%) had been hospitalized in a room with air treatment; of these, 100 (95%) were hospitalized in a laminar flow sterile room or IMMUNAIR™ bed, or in a highly purified HEPA-filtered room. Time since entry into the unit was different when split by strategy type: seven (64%) patients who received pre-emptive or curative therapy had entered the unit for ≥ 30 days or more, compared with 20 (21%) patients in the antifungal prophylaxis group and three (23%) patients in the empiric therapy group.

**Table 5:** Characteristics of hospitalized patients treated according to different systemic antifungal strategies, during the 5-day observation period

	Prophylactic strategy (*n* = 95)	Empiric strategy (*n* = 13)	Pre-emptive or curative strategy (*n* = 11)	Total (*N* = 119)
Sex: male	56 (59)	6 (46)	5 (45)	67 (56)
Adults	88 (93)	11 (85)	11 (100)	110 (92)
*Median age (year)*				
Adult (range)	52.2 (21 − 71)	40.7 (28 − 63)	52.9 (22 − 64)	51.4 (21 − 71)
Children (range)	7.7 (0 − 12)	12.4 (12 − 12)		7.9 (0 − 12)
*Hematological malignancy*				
Acute myeloid leukemia	41 (43)	6 (46)	8 (73)	55 (46)
Myelodysplastic syndrome	5 (5)	1 (8)	1 (9)	7 (6)
Acute lymphoblastic leukemia	16 (17)	2 (15)	1 (9)	19 (16)
Hodgkin lymphoma	9 (9)	1 (8)	1 (9)	11 (9)
Non-Hodgkin lymphoma	9 (0)	0	0	9 (8)
Chronic lymphoid leukemia	1 (1)	0	0	1 (1)
Myeloma	5 (5)	1 (8)	0	6 (5)
Chronic myeloid leukemia	3 (3)	2 (15)	0	5 (4)
Other	6 (6)	0	0	6 (5)
Disease status: relapse or refractory	56 (59)	8 (62)	10 (91)	74 (62)
*Underlying conditions*				
GVHD^a,b^	27 (28)	3 (23)	6 (55)	36 (30)
Grade I − II acute GVHD	10 (37)	1 (33)	5 (83)	16 (44)
Grade III − IV acute GVHD	14 (52)	1 (33)	1 (17)	16 (44)
Chronic GVHD	7 (26)	2 (67)	1 (17)	10 (28)
Neutropenic phase	38 (40)	9 (69)	4 (36)	51 (43)
Neutropenia for at least 10 days	16 (17)	9 (69)	4 (36)	29 (24)
Persistent fever refractory to antibiotic therapy	6 (6)	5 (38)	3 (27)	14 (12)
Previous IFD	19 (20)	2 (15)	4 (36)	25 (21)
*Ongoing treatments*				
Chemotherapy	44 (46)	6 (46)	4 (36)	54 (45)
Antibiotics	80 (84)	12 (92)	11 (100)	103 (87)
Immunosuppressors	67 (71)	13 (100)	7 (64)	87 (73)
Antivirals	87 (92)	11 (85)	9 (82)	107 (90)
*Time since entry in the unit*				
≥ 30 days	20 (21)	3 (23)	7 (64)	30 (25)
< 15 days	61 (64)	3 (23)	3 (27)	67 (56)
Hospitalization in a room with air treatment	82 (86)	13 (100)	10 (91)	105 (88)
Laminar flow sterile room or IMMUNAIR™ bed^c^	60 (73)	11 (85)	8 (80)	79 (75)
Highly purified HEPA-filtered room^c^	18 (22)	1 (8)	2 (20)	21 (20)
Conventional room^c,d^	4 (5)	1 (8)	0	5 (5)

Data are *n* (%), unless otherwise specified

GVHD: graft-versus-host disease; HEPA: high-efficiency particulate air; IFD: invasive fungal disease

^a^Chronic and/or acute GVHD

^b^Percentage among patients with GVHD

^c^Percentage among patients hospitalized in room with air treatment

^d^Room with PLASMAIR™ or equivalent

Of the 95 patients who received antifungal prophylaxis, 56 (59%) were in relapse or refractory disease and 38 (40%) were neutropenic. Twenty-seven (28%) patients presented GVHD; of these, 14 (52%) had acute grade III−IV GVHD, 10 (37%) had acute grade I−II GVHD, and seven (26%) had chronic GVHD. Nineteen (20%) patients had a history of IFD.

Of the 13 patients who received empiric therapy, eight (62%) had relapse or refractory disease and two (15%) had a history of IFD. Nine (69%) patients were neutropenic, all of whom presented neutropenia for at least 10 days.

Eleven patients were treated according to a pre-emptive or curative strategy; most (*n* = 10; 91%) were in relapse or refractory disease and four (36%) had already experienced an IFD episode. Four (36%) patients were neutropenic and six (55%) had GVHD with five (83%) acute grade I–II GVHD.

## Discussion

The AFHEM study was the first cross-sectional observational study to determine the use of antifungal treatment strategies in alloHSCT recipients in clinical practice in France. It is widely acknowledged that routine administration of systemic antifungal prophylaxis in the alloHSCT setting significantly reduces both the incidence of probable and proven IFD, and of IFD-related mortality [15, 16]. In our study, the rate of alloHSCT recipients receiving antifungal prophylaxis was higher than in the overall population of the global AFHEM study (95/147 [65%] for the alloHSCT population compared with 187/494 [38%] for the rest of the studied population in the global AFHEM study).

Azoles (mainly fluconazole) were the drugs preferentially administrated to prevent invasive candidiasis, combined with sterile air conditions in order to prevent IFD due to filamentous fungi. Caspofungin was the most commonly used echinocandin for prophylaxis in alloHSCT recipients (*n* = 11; 85%), although it is not licensed for this indication. Although a systematic review published in 2014 failed to demonstrate consistent treatment effects of antifungal prophylaxis for IFD-related mortality and IFD incidence in alloHSCT recipients [17], the AFHEM study showed that prophylaxis was the leading strategy used in alloHSCT recipients. Following the publication of several international guidelines since 2010, the use of antifungal prophylaxis has increased in clinical practice [14, 16, 18–21], especially in high-risk patients. Moreover, the development of new broad-spectrum antifungals has led to their use as prophylactic agents rather than delaying treatment until clinical signs of infection manifest [22].

Considering patients in the pre-engraftment period and patients developing GVHD, most (*n* = 85; 89%) had received systemic antifungal therapy, especially those presenting with a high-risk profile according to ECIL-5 and ECIL-6 criteria (i.e., including acute myeloid leukemia, refractory or relapsed acute lymphoblastic leukemia, age over 65, history of IFD, and acute GVHD) [23].

Although a period of severe neutropenia is reportedly the most important risk factor for IFD in patients receiving standard chemotherapy for hematologic malignancies [9, 15], several studies have shown that most IFD cases are reported more than 100 days after alloHSCT, once full hematologic recovery has occurred [9, 24–27]. In the primary analysis of the AFHEM study, approximately half of alloHSCT patients had neutropenia, with 50% of these patients in the early neutropenic phase (lasting less than 10 days), which is consistent with the high level of prophylaxis observed in our subgroup analysis, considering the high level of risk for antifungal disease conferred by neutropenia.

Antifungal prophylaxis in alloHSCT recipients with GVHD is a practice that is currently supported by findings from randomized controlled trials and the recommendations of international guidelines [16–20, 23, 28–31]. However, factors that influence the selection of antifungals for prophylaxis remain complex and should probably be dictated by local epidemiology, hospital-specific logistics, and risk stratification based on the profile of different subpopulations of patients receiving alloHSCT [5]. Moreover, antifungal prophylaxis strategies should never replace the appropriate management of infection control nor the implementation of patient education strategies to avoid exposure to invasive fungal agents, especially in patients with long-term immunosuppression [5]. Notably, late-invasive aspergillosis has emerged as a concern in patients receiving continual immunosuppression for chronic GVHD. In our study, only one-third of patients had GVHD (either acute and/or chronic) but 70% were receiving immunosuppressive treatment, and more than 85% received antifungal medication, mainly as prophylaxis or empiric therapy, while ECIL recommends that patients with GVHD should receive immunosuppressors together with antifungal prophylaxis, but no empiric therapy.

This study had several limitations. Although this was a prospective study, its cross-sectional design did not consider the development of antifungal strategies according to the changing clinical conditions. Due to its observational nature, the antifungal strategies (i.e. prophylaxis, empiric strategy, and pre-emptive or curative strategies) were recorded according to the physicians’ judgement and may not match the current definitions published in existing guidelines. The AFHEM study was conducted several years ago in 2013, however, there have been few changes to clinical practice since this time; for instance, the latest European guidelines for primary antifungal prophylaxis still recognize azoles, and in particular fluconazole, as the primary antifungal prophylaxis for use in patients with alloHSCT [14], which is consistent with the findings of this report. Finally, the results are limited to patients enrolled from French centers that agreed to participate in the study and, as such, they may be not representative of the overall use of antifungal therapy in France.

## Conclusions

This work provides important data on the antifungal strategies used in alloHSCT recipients hospitalized in French hematological units. Prophylaxis is now the leading antifungal strategy used in these patients, with 80% of alloHSCT recipients treated in this way throughout the AFHEM study. This analysis gives further support that an antifungal stewardship program may lead to improved clinical outcomes.

## Data Availability

Access to anonymized individual participant level data will not be provided for this trial as it meets one or more of the exceptions described on www.clinicalstudydatarequest.com under “Sponsor Specific Details for Astellas.”

## Abbreviations

alloHSCT: Allogeneic hematopoietic stem cell transplantation
ECIL: European Conference on Infections in Leukemia
GVHD: Graft-versus-host disease
HEPA: High-efficiency particulate air
IFD: Invasive fungal diseases

## References

[1] Mikolajewska A, Schwartz S, Ruhnke M (2012). Antifungal treatment strategies in patients with haematological diseases or cancer: from prophylaxis to empirical, pre-emptive and targeted therapy. Mycoses.

[2] Strasfeld L, Weinstock DM (2006). Antifungal prophylaxis among allogeneic hematopoietic stem cell transplant recipients: current issues and new agents. Expert Rev Anti Infect Ther.

[3] Parameswaran GI, Segal BH, Almyroudis NG (2008). Antifungal agents in hematopoietic stem cell transplantation. Curr Pharm Des.

[4] McCoy D, Depestel DD, Carver PL (2009). Primary antifungal prophylaxis in adult hematopoietic stem cell transplant recipients: current therapeutic concepts. Pharmacotherapy.

[5] Kontoyiannis DP (2011). Antifungal prophylaxis in hematopoietic stem cell transplant recipients: the unfinished tale of imperfect success. Bone Marrow Transplant.

[6] Bow EJ (2005). Long-term antifungal prophylaxis in high-risk hematopoietic stem cell transplant recipients. Med Mycol.

[7] Marr KA, Crippa F, Leisenring W (2004). Itraconazole versus fluconazole for prevention of fungal infections in patients receiving allogeneic stem cell transplants. Blood.

[8] Cornely OA, Ullmann AJ, Karthaus M (2003). Evidence-based assessment of primary antifungal prophylaxis in patients with hematologic malignancies. Blood.

[9] Martino R, Subira M, Rovira M (2002). Invasive fungal infections after allogeneic peripheral blood stem cell transplantation: incidence and risk factors in 395 patients. Br J Haematol.

[10] Gangneux JP, El Cheikh J, Herbrecht R (2018). Systemic antifungal prophylaxis in patients hospitalized in hematology units in France: the AFHEM cross-sectional observational study. Infect Dis Ther.

[11] El-Cheikh J, Venton G, Crocchiolo R (2013). Efficacy and safety of micafungin for prophylaxis of invasive fungal infections in patients undergoing haplo-identical hematopoietic SCT. Bone Marrow Transplant.

[12] El Cheikh J, Ceballos P, Dalle JH (2019). Micafungin prophylaxis in routine medical practice in adult and pediatric patients with hematological malignancy: a prospective, observational study in France. Diagn Microbiol Infect Dis.

[13] De Pauw B, Walsh TJ, Donnelly JP (2008). Revised definitions of invasive fungal disease from the European Organization for Research and Treatment of Cancer/Invasive Fungal Infections Cooperative Group and the National Institute of Allergy and Infectious Diseases Mycoses Study Group (EORTC/MSG) Consensus Group. Clin Infect Dis.

[14] Maertens JA, Girmenia C, Brüggemann RJ, et al. European guidelines for primary antifungal prophylaxis in adult haematology patients: summary of the updated recommendations from the European Conference on Infections in Leukaemia. J Antimicrob Chemother. 2018;73:3221-30. 10.1093/jac/dky286 30085172

[15] Omer AK, Ziakas PD, Anagnostou T (2013). Risk factors for invasive fungal disease after allogeneic hematopoietic stem cell transplantation: a single center experience. Biol Blood Marrow Transplant.

[16] Bow EJ, Vanness DJ, Slavin M (2015). Systematic review and mixed treatment comparison meta-analysis of randomized clinical trials of primary oral antifungal prophylaxis in allogeneic hematopoietic cell transplant recipients. BMC Infect Dis.

[17] Ziakas PD, Kourbeti IS, Mylonakis E (2014). Systemic antifungal prophylaxis after hematopoietic stem cell transplantation: a meta-analysis. Clin Ther.

[18] Mousset S, Buchheidt D, Heinz W (2014). Treatment of invasive fungal infections in cancer patients-updated recommendations of the Infectious Diseases Working Party (AGIHO) of the German Society of Hematology and Oncology (DGHO). Ann Hematol.

[19] Girmenia C, Aversa F, Busca A (2013). A hematology consensus agreement on antifungal strategies for neutropenic patients with hematological malignancies and stem cell transplant recipients. Gruppo Italiano Malattie Ematologiche dell’Adulto, Gruppo Italiano Trapianto di Midollo Osseo, Associazione Italiana Ematologia ed Oncologia Pediatrica, Invasive Fungal Infections Cooperative Group of the European Organization for Research and Treatment of Cancer and Sorveglianza Epidemiologica delle Infezioni Fungine nelle Emopatie Maligne. Hematol Oncol.

[20] Ullmann AJ, Akova M, Herbrecht R (2012). ESCMID* guideline for the diagnosis and management of Candida diseases 2012: adults with haematological malignancies and after haematopoietic stem cell transplantation (HCT). Clin Microbiol Infect.

[21] Maertens J, Marchetti O, Herbrecht R (2011). European guidelines for antifungal management in leukemia and hematopoietic stem cell transplant recipients: summary of the ECIL 3–2009 update. Bone Marrow Transplant.

[22] Almyroudis NG, Segal BH (2010). Antifungal prophylaxis and therapy in patients with hematological malignancies and hematopoietic stem cell transplant recipients. Expert Rev Anti Infect Ther.

[23] Tissot F, Agrawal S, Pagano L (2017). ECIL-6 guidelines for the treatment of invasive candidiasis, aspergillosis and mucormycosis in leukemia and hematopoietic stem cell transplant patients. Haematologica.

[24] Junghanss C, Marr KA, Carter RA (2002). Incidence and outcome of bacterial and fungal infections following nonmyeloablative compared with myeloablative allogeneic hematopoietic stem cell transplantation: a matched control study. Biol Blood Marrow Transplant.

[25] Fukuda T, Boeckh M, Carter RA (2003). Risks and outcomes of invasive fungal infections in recipients of allogeneic hematopoietic stem cell transplants after nonmyeloablative conditioning. Blood.

[26] Labbé AC, Su SH, Laverdière M (2007). High incidence of invasive aspergillosis associated with intestinal graft-versus-host disease following nonmyeloablative transplantation. Biol Blood Marrow Transplant.

[27] Ben-Ami R, Lewis RE, Kontoyiannis DP (2009). Invasive mould infections in the setting of hematopoietic cell transplantation: current trends and new challenges. Curr Opin Infect Dis.

[28] Robenshtok E, Gafter-Gvili A, Goldberg E (2007). Antifungal prophylaxis in cancer patients after chemotherapy or hematopoietic stem-cell transplantation: systematic review and meta-analysis. J Clin Oncol.

[29] Walsh TJ, Anaissie EJ, Denning DW (2008). Treatment of aspergillosis: clinical practice guidelines of the Infectious Diseases Society of America. Clin Infect Dis.

[30] Tomblyn M, Chiller T, Einsele H (2009). Guidelines for preventing infectious complications among hematopoietic cell transplantation recipients: a global perspective. Biol Blood Marrow Transplant.

[31] Cornely OA, Böhme A, Buchheidt D (2009). Primary prophylaxis of invasive fungal infections in patients with hematologic malignancies. Recommendations of the Infectious Diseases Working Party of the German Society for Haematology and Oncology. Haematologica.

